# An Innovative Educational Model in Intraocular Pressure Measurement

**Published:** 2012

**Authors:** Roghayeh Heidary, Fatemeh Heidary, Abolfazl Rahimi, Reza Gharebaghi

**Affiliations:** 1 Alborz University of Medical Sciences, Karaj, Iran,; 2 Shahid Beheshti University of Medical Sciences,; 3 Dept. of Ophthalmology, Tehran Medical Branch, Islamic Azad University,; 4 Editorial Office, Medical Hypothesis, Discoveries and Innovation Ophthalmology Journal

**Keywords:** Tactile palpation, Intraocular pressure, Educational model, Glaucoma

## Abstract

Tactile palpation is a simple technique that can prove useful for estimating intraocular pressure (IOP) in primary healthcare settings, mainly in the absence of equipment, for very young children, patients who are intellectually challenged, those with eyes with extremely irregular corneas, and patients with corneal prostheses. Accordingly, this technique can also aid in the diagnosis of high IOP in primary and emergency care settings. To the best of our knowledge, there is no instrument that can quantify the estimation of IOP and teach tactile examiners. This group has developed a digital instrument called the MEHDI-IOP Measurement Model to train primary healthcare workers as well as blind individuals in the estimation of IOP. In this simple instrument, elastic spheres with a specific inner pressure can be touched and the responses of candidates with regard to the estimated pressure can be graded accordingly.

## INTRODUCTION

Glaucoma is a multifactorial optic neuropathy in which there is a characteristic acquired loss of retinal ganglion cells and corresponding atrophy of the optic nerve. Although asymptomatic in its earlier stages, the disease is nonetheless one of the foremost global causes of irreversible blindness [1]. It is considered one of the leading causes of blindness worldwide and twelve million individuals globally are estimated to be blind because of the disease [2]. 

Elevated IOP is still the major risk factor for an untreated glaucomatous eye to progress to a more severe stage of glaucoma. Three methods of evaluating the IOP exist: palpation, manometry, and tonometry. Indentation, applanation, contour matching, and rebound are the main physical principles of tonometers that are applicable in the clinical setting at present [1]. 

**Importance of digital palpation in IOP measurement**

Palpation is one of the simplest and least expensive techniques for approximate IOP assessment. The estimation of IOP by digital palpation through the upper eyelid dates back a long time. Despite a few supporter studies, digital palpation has not been successful when put to the test. However, in most current practices, digital palpation is used for those conditions where tonometry is not possible. For example, those who are intellectually challenged, have eyes with enormously irregular corneas, infants and young children, as well as those who have corneal prostheses [4]. 

Based on successful findings of a study that used visually impaired individuals as breast examiners, it has already been hypothesised that blind individuals could also serve as examiners for the estimation of IOP using tactile palpation [5]. Moreover, it is also recommended that primary health workers routinely perform digital palpation on patients, and assess the accuracy of their findings using conventional tonometers. With minimal training, individuals may improve their expertise with the practice and the accuracy of their estimation may be enhanced. To the best of our knowledge, there is no instrument that can quantify the estimation of IOP and teach tactile examiners. 

## HYPOTHESES AND DISCUSSION

**MEHDI-IOP Measurement Model**

**Figure 1:**
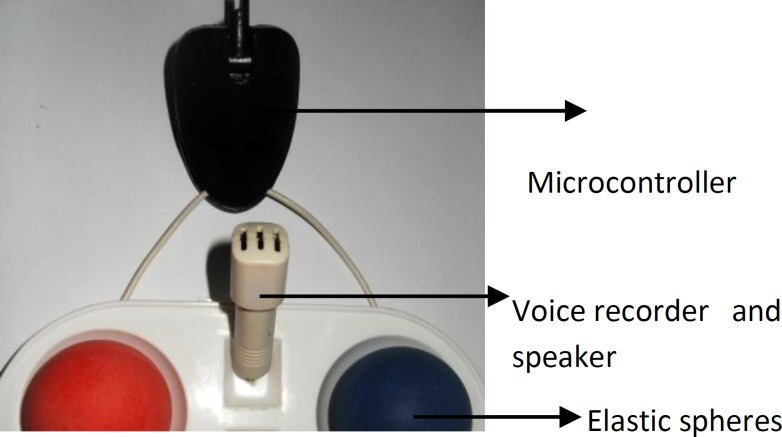
Components of MEHDI-IOP Measurement Model

This model can be used to train primary healthcare workers as well as blind individuals for the estimation of IOP. In this simple innovative instrument, elastic spheres with a specific inner pressure ranging from 5 to 100mmHg could be touched and the responses of candidates recorded by a voice recorder, with regard to the estimated pressure. Since the examiner may be selected from blind individuals, a voice alarm is implanted in the instrument to inform the examiner of the estimation accuracy. Materials used in the device, such as a non-flammable, low-toxicity waterproof coating, and non-allergy-inducing materials, may protect the integrity of the instrument even in a wet environment. Other practical advantages of this device are its small size, low weight, and ability to connect with a computer. The instrument consists of a voice recorder, speaker, microcontroller, rechargeable battery and external port covered with shock-absorbent material for eliminating unwanted and excess motion to protect incidental trauma. Data can be stored in a memory and read out after recording by connecting it to a PC or mobile. Fig. 1 shows the components of the MEHDI-IOP Measurement Model. In general, examiners who are at least 95% successful in determining accuracy can receive certificates as Health Tactile Examiners. The license should be renewed after a certain amount of time has passed.

## CONCLUSION

In summary, this portable and inexpensive instrument could be used to standardise digital palpation and may assist in teaching tactile examiners. More studies are warranted to justify its clinical use. 

## DISCLOSURE

The authors report no conflicts of interest in this work. 
